# The Nasal Treatment of Asthma

**Published:** 1902-10-25

**Authors:** 


### THE NASAL TREATMENT OF ASTHMA.

At the opening meeting of the Clinical Society the subject discussed raised many questions which perhaps are hardly worth theorising about until we can be more sure of the facts, at least as they exist in this climate, than seemed to be admitted by some of the speakers. Dr. Alexander Francis, of Brisbane, made a communication on the nasal treatment of asthma. He said that most rhinologists had faith in the nasal treatment of asthma where obvious trouble existed, but none had suggested the advisability of treating the nose in cases of asthma where the organ was apparently normal; yet in Dr. Francis's opinion such were the cases where the most hopeful prognosis could be given. Of 402 cases recorded, 346 had no apparent nasal lesion, and of these only eight obtained no relief from nasal treatment, while six cases were unrelieved by treatment among 56 which had polypi or other gross nasal lesion. In 17 cases the result of treatment could not be ascertained. It was concluded then: 1. That asthma was due to reflex spasm of the bronchial tubes. 2. That the irritation might originate in the nose. 3. That asthma was not directly due to any mechanical obstruction of the nasal passages and was not commonly caused by any gross nasal lesion. The association of asthma with polypi was not so common as was generally supposed, but where polypus had been present in cases of asthma the best results had been obtained by cauterising the septum without touching the polypi, whereas in some cases complete eradication of the polypi had intensified the asthamatic condition. In other cases again, where nasal obstruction from engorged turbinates and asthma occurred together, the difficulty of breathing through the nose and the dyspnœa were unrelieved by rendering the nasal passages mechanically free, but were both instantly removed on applying cocaine to the septum. 4. That some part of the nasal apparatus had a controlling influence on the respiratory centre. Now all this seemed very subversive of a good deal that has of late been laid down with a certain degree of positiveness both as to the nature of asthma and of the manner in which nasal treatment gives relief in certain cases, and several members of the society expressed considerable astonishment at the results obtained. Dr. Scanes Spicer was surprised at the large percentage of lasting successes claimed, as he understood, from galvano-cautery treatment alone. In that respect his own experience, gained in this country and in cases of asthmatics who mostly had marked nasal symptoms, did not coincide with the author's. An appreciable fraction of his own cases had received great and long standing benefit from cautery alone, but usually he had found the nasal abnormalities of a much more complex and structural character than could be dealt with by that means. Dr. Herbert Tilley said that the statistics and statements in the author's paper were astounding. The meeting of the Laryngcological Society was attended by physicians and surgeons of London, Edinburgh, and other cities, and yet only nine cases of asthma were recorded at that meeting as "cures," or as obtaining very great relief from intranasal treatment. Perhaps the air of Australia tended to make this treatment more efficacious than it was in England.

